# Association analysis of Suboptimal health Status: a cross-sectional study in China

**DOI:** 10.7717/peerj.10508

**Published:** 2020-12-11

**Authors:** Yunlian Xue, Zhuomin Huang, Guihao Liu, Yefang Feng, Mengyao Xu, Lijie Jiang, Jun Xu

**Affiliations:** 1 Department of Sanitation Economy Administration, Nanfang Hospital, Southern Medical University, Guangzhou, Guangdong Province, China; 2 Guangdong Provincial People’s Hospital (Guangdong Academy of Medical Sciences), Guangzhou, Guangdong Province, China; 3 School of Public Health, Southern Medical University, Guangzhou, Guangdong, China; 4 School of Health Services Management, Southern Medical University, Guangzhou, Guangdong Province, China

**Keywords:** Suboptimal health, Health promotion, Urban residents, Influencing factors model

## Abstract

**Background:**

Suboptimal health status (SHS) among urban residents is commonplace in China. However, factors influencing SHS have not been thoroughly explored, especially with regard to the effects of internal factors (e.g., personality and health awareness) on SHS.

**Methods:**

A cross-sectional study was conducted with a nationally representative sample of 5460 Chinese urban residents..SHS was measured using the Suboptimal Health Mesurement Scale Version 1.0. Demographic information, and information pertaining to lifestyle behaviors, environmental factors, and internal factors were abtained through a questionnaire. The associations between demographic information, lifestyle behaviors, environmental factors, internal factors and SHS were assessed using logistic regression.

**Results:**

Of the 5460 participants (with a mean age of 41.56 ±  16.14 years), 2640 (48.4 %) were men. Out of 36 variables, 23 were significantly associated with SHS: age (odds ratio [OR]: 1.014), an education level of high school/junior college (OR: 1.443) , marital status (OR: 1.899), area of registered permanent residence (OR: 0.767), monthly household income (*p* < 0.001) , exposure to second-hand smoke (*p* = 0.001), alcohol drinking (OR: 1.284), bad eating habits (OR: 1.717), not sleeping before 11 p.m. every day (*p* = 0.002), spending time online more than five hours a day (OR: 1.526), having a good relationship with parents during one’s growth period (OR: 0.602), living with good quality air (OR:0.817), living in not crowded conditions (OR:0.636), having a harmonious neighborhood (OR:0.775), having adequate fitness facilities (OR:0.783), one’s health being affected by two-child policy (OR: 1.468) and medical policies (OR: 1.265) , high adverse quotient (OR: 0.488), many (≥3 kinds) interests and hobbies (OR: 0.617), mature and steady personality traits (OR: 0.469) , a high attention to one’s health (OR: 0.833), and effective health promotion induced by leading a leisurely lifestyle (OR: 0.466) were significantly associated with SHS.

**Conclusions:**

All these variables were included demographic information, lifestyle behaviors, environmental factors and internal factors. Our study supports the benefits of controlling both internal and external factors in preventing suboptimal health.

## Introduction

Suboptimal health status (SHS) is the “third state” between health and disease, carrying the characteristics of transformation from health to disease. According to a 2013 global survey by the World Health Organization (WHO), only 5% of people could be classified as truly healthy, 20% were sick, and people in SHS was accounted in 75% of people (Ma & Zhu, 2013). In addition to acquired immunodeficiency syndrome, hazards that result from SHS have been recognized in the medical field as being of top concern in the 21st century (Zhao, 2006). If SHS is properly handled, the human body can be transformed into a healthy state, or turn the disease the other way around (Chen et al., 2014). It is therefore of significance to explore the factors influencing SHS for its prevention and intervention.Many individuals may not be aware that they are suffering from SHS. For instance, a 1998 study with 6,000 asymptomatic “healthy people” indicated that 72.8% of these people were in the “suboptimal health status” range (Liu & Li, 2001) . The identification of SHS is the key to preventing the deterioration of an individual’s health status. SHS can reportedly be measured objectively using the microbiome (Sun et al., 2020), telomere length (Alzain et al., 2017), plasma stress hormones (Yan et al., 2018), plasma metabolites (Wang et al., 2020), glycan (Adua et al., 2019a; Adua et al., 2019b; Wang & Tan, 2019), ideal cardiovascular health metrics (Wang et al., 2017), and traditional Chinese medicine (Wang & Yan, 2012; Wang, Russell & Yan, 2014). However, these objective measures are not easily accessible, and may sometimes not be obvious, especially when people feel uncomfortable and are without abnormal symptoms. Self-rated assessment using a questionnaire has been shown to be widely applicable for assessing SHS, with the first Chinese study on suboptimal health measured using a questionnaire being published in the English literature in 2009 (Yan et al., 2009).

In China, the Sub-Health Measurement Scale (SHMS V1.0) (Xue et al., 2019a; Xue et al., 2019b; Xue et al., 2019c; Xu et al., 2012; Xie et al., 2016; Lu et al., 2013), suboptimal health status questionnaire (SHSQ-25) (Yan et al., 2011; Adua, Roberts & Wang, 2017; Kupaev et al., 2016; Hou et al., 2018; Anto et al., 2019; Adua et al., 2019a; Adua et al., 2019b) and Chinese sub-health scale (CSHES) (Xu et al., 2012) have been widely used for assessing SHS. However, unlike other questionnaires, the SHMS V1.0 assesses of the physical, mental, and social aspects of SHS, which is in accordance with the concept of health proposed by the WHO in 1947.

With the rapid urbanization in China in recent years, the lifestyle and living environment of urban residents have changed substantially (Chen et al., 2017a; Chen et al., 2017b). However, urbanization is a double-edged sword for urban residents’ health (Li et al., 2016). On the one hand, to some extent, urbanization has improved the health benefits of urban residents due to easier access to health care services and knowledge than that available to the rural population. On the other hand, risk factors, such as air pollution, having an unhealthy diet, living a sedentary lifestyle and the life pressure (Miao & Wu, 2016) of chronic diseases were increased for urban residents. The first SHS study that involved urban Chinese population indicated (Yan et al., 2012) that SHS was associated with risk factors of chronic diseases and contributed to the development of them. Previous studies have shown that lifestyle behaviors are some of the most important contributing factors including smoking, alcohol consumption, irregular breakfast habits, malnutrition, lack of exercise, and sleeping problems (Lolokote, Hidru & Li, 2017; Chen et al., 2017a; Chen et al., 2017b). Drinking, inadequate breakfast, sleep quality and negative event experience have been reported to be significantly associated with suboptimal health for urban residents (Xie et al., 2016). The environment can also greatly influences one’s overall health (Messer, Maxson & Miranda, 2013). As such, SHS can be aggravated by both air pollution (Kelly & Fussell, 2015) and noise (Clark & Paunovic, 2018).

Despite all the above, previous studies on the association factors of SHS for urban residents have only investigated specific aspects, especially external factors, such as lifestyle and environmental factors. Internal factors, such as personality characteristics, and health consciousness have rarely been studied. Materialist dialectics holds that the development of things is the result of both internal and external factors: that the external cause influences the development of things through internal factors. Thus, this study attempts to determine the factors associated with internal factors, such as personality characteristics and health consciousness, and external factors, such as lifestyle and environmental factors, among urban residents in China with suboptimal health.

## Materials and Methods

### Study setting and participants

Multi-stage stratified sampling method was used to select participants. For the first stage, according to the administrative regions division and economic development levels in China, one province or city was selected randomly from the following regions: North, Northeast, South-Central, Southwest and Northwest. The selected provinces and cities were Tianjin City, Harbin City, Guangdong Province, Sichuan Province, and Lanzhou City. For the second stage, extracted three to four cities or districts were identified based on the five selected provinces and cities in the first stage while considering their economic level and geographical distributions. For the third stage, one to four districts/streets that were administratively under the identified cities and districts from second stage were selected randomly. For the fourth stage, one to two communities in the selected streets from the third stage were chosen. Qualified participants were then investigated. Sex and age were also considered in the recruitment, under the standard of male: female = 1:1 for sex and (<24):(25–34):(35–44):(45–54):(55–64):(>65) = 1:1:2:2:1:1 for age.

Participants had to be more than 14 years old, had lived in the cities or towns for more than half a year, provided verbal informed consent, and voluntarily participated in the survey. The data of those who could not complete the questionnaire due to vision or hearing impairment or other diseases were excluded from the analysis. Urban residents who were ≥14 years old in the identified communities from the third stage were identified as study participants. A total of 7,293 questionnaires were distributed and 6,748 valid questionnaires were collected. Finally, we investigated 1,150, 1,538, 1,898, 1,084 and 1,078 participants in Tianjin City, Haerbin City and Lanzhou city, Guangdong Province and Sichuan Province, respectively The number of participants surveyed in each region met the minimum sample size requirements that was calculated according to the sub-health detection rate in each region (961 in Guangdong Province, 940 in Lanzhou City, 946 in Harbin City, 956 in Sichuan Province, and 928 in Tianjin City).Based on the sampling method and sample size, the overall sample population in this study is broadly representative of urban residents in China. This study was approved by the medical ethics committee of Nanfang Hospital of Southern Medical University (No. NFEC-2019-196). All data collected were kept strictly confidential.

### Suboptimal health status

SHS was the health outcome analyzed in this study. This was measured using the Suboptimal Health Measurement Scale version 1.0 (SHMS V1.0) , which had been previously developed by our research group (Sun et al., 2008; Xie et al., 2016; Lu et al., 2013), and has been widely used for SHS assessment and found to be significantly reliable (Xu et al., 2019). The SHMS V1.0 comprises 39 items (SH1-SH39), each including five response, asking the participants to choose the options that are the closest to their actual feelings in the past four weeks. The SHMS V1.0 scale encompasses three sub-scales with respect to physiological suboptimal health (SH1-SH14), psychological suboptimal health (SH16-SH27) and socially suboptimal health (SH29-SH37), whose scores are marked as PS, MS, and SS, respectively. The overall score of the three sub-scales is the score (marked as GS) of the whole scale. Higher scores indicate better health. According to the threshold norm of the SHMS V1.0 scale for urban residents in China (Table 1) established according to gender and age in our previous study (Xu et al., 2019), the health status for Chinese urban residents can be divided into three states with respect to disease, SHS (including severe, moderate, and mild SHS) and health.

### Covariates

A total of 36 variables were investigated for the association analysis, with the pertinent data elicited from participants using a self-completed questionnaire. All variables were summed up into four parts: demographic information, lifestyle behaviors, environmental factors, and internal factors.

Demographic information included the participants’ registered permanent residence, which was categorized as either urban or rural; educational level, which was categorized as either junior school and below, high school/junior college, or university and above; marital status, which was categorized as either married, unmarried, divorced, or widowed; and monthly household income per capita (¥), which was categorized as either <¥2,500, ¥2,500–5,000, ¥5,000–7,500, ¥7,500–10,000, or above ¥10,000.

**Table 1:** Threshold norm of SHMS V1. Zero, general score in Chinese urban residents.

Gender	Age	Disease	Sever SHS	Moderate SHS	Mild SHS	Health
Men	14–19	[0, 57.89)	[57.89, 64.16)	[64.16, 76.7)	[76.7, 82.96)	[82.96, 100]
	20–29	[0, 56.64)	[56.64, 62.47)	[62.47, 74.13)	[74.13, 79.96)	[79.96, 100]
	30–49	[0, 55.85)	[55.85, 62.19)	[62.19, 74.85)	[74.85, 81.18)	[81.18, 100]
	50–64	[0, 54.98)	[54.98, 61)	[61, 73.05)	[73.05, 79.08)	[79.08, 100]
	≥65	[0, 52.89)	[52.89, 59.16)	[59.16, 71.7)	[71.7, 77.97)	[77.97, 100]
Women	14–19	[0, 55.21)	[55.21, 61.54)	[61.54, 74.21)	[74.21, 80.55)	[80.55, 100]
	20–29	[0, 56.01)	[56.01, 61.76)	[61.76, 73.24)	[73.24, 78.99)	[78.99, 100]
	30–49	[0, 54.95)	[54.95, 60.99)	[60.99, 73.05)	[73.05, 79.09)	[79.09, 100]
	50–64	[0, 54.37)	[54.37, 60.37)	[60.37, 72.37)	[72.37, 78.37)	[78.37, 100]
	≥65	[0, 51.71)	[51.71, 57.93)	[57.93, 70.36)	[70.36, 76.57)	[76.57, 100]

Lifestyle behaviors include smoking status, which was categorized as either nonsmoker, past smoker (smoked in the past and quit a month ago), or smoker; exposure to secondhand smoke, which was categorized as never, occasionally, sometimes, or often; alcohol drinking, which was categorized as either yes or no; breakfast habit, which was defined as the frequency of having breakfast, which was categorized as either every day, often, sometimes, occasionally, or never; bad eating habits, which included irregular eating, poor dieting, overeating, partial or picky eating, high intake of salt, eating spicy food, and often eating snacks instead of regular meals, which were categorized as either yes or no; falling asleep before 11 p.m., which was categorized as either every day, often (3–6 days/week), or rarely (≤2 days/week); spending time online, which was categorized as either <1 h, 1 h, 3 h, or ≥5 h; basking in the sun, which was categorized as either ≥7 h, 3 h, 1 h, or <1 h; and physical exercise, which was categorized as either every day, often (5–6 days/week), sometimes (3–4 days/week), or occasionally (<2 days/week).

Meanwhile, environmental factors include the relationship with parents during one’s growth period, which was categorized as either good or bad; living with good quality air quality, living with plentiful greenery; living in simple or crude houses, living in crowded conditions, living in a harmonious neighborhood having access to adequate fitness facilities; having access to adequate medical and educational resources; living together with family; one’s health affected by medicine policies and one’s health being affected by the two-child policy, which were all categorized as either yes or no; family structure, which was categorized as either incomplete, nuclear, or extended family; and parents’ discipline, which was categorized as either spoiled, stern, or positive.

Finally, internal factors included adverse quotient, which was categorized as either low or high; interests and hobbies, which were categorized as either few (<3) or many (≥3); personality traits, which were categorized as either anxiety and irritability, swallowing rage and suffering in silence, impatient and aggressive, or mature and steady; attending health training and lectures, which was categorized as either occasionally, sometimes, or often; health promotion exerted by leading a leisurely lifestyle, which was categorized into moderate or mild, or good; life attitude, which was categorized as either negative or positive; being ware of the importance of interpersonal communication, which was categorized as either unimportant, general, or important and attention to one’s own health, which was categorized as either low or high.

### Statistical analysis

The software SPSS20.0, R and AMOS23 were used for the statistical analysis. Using percentage for the descriptions of count data, we conducted a comparison using chi-square test. Quantitative data were described as ($\overline{X}\pm S$) and analyzed using *t*-test. A generalized linear mixed model (GLMM) was used to calculate the intra-class correlation coefficient (ICC) and examine the cluster effect of the sampling area. Logistic regression was used to analyze the influencing factors, which turned out to have statistically significant difference (*p* < 0.05).

## Results

The SHS incidence rate of urban residents in the five investigated provinces and cities in China was 66.7%. The average age of these urban residents was 41.56 ±16.14, of which 2640 (48.4%) were men and 2820 (51.6%) were women. The descriptive analysis and comparison by gender are presented in Table 2. Females were more likely to be divorced and widowed, have a monthly household income per capita less than ¥5000/month, have a higher body mass index, be a nonsmoker, have never exposed to secondhand smoke, not drink alcohol, have breakfast every day, bask in the sun less than three hours a week, occasionally do physical exercise, fall asleep before 11 p.m. (day/week) every day, live together with family, have parents who practiced disciplining, live with plentiful greenery, live in a harmonious neighborhood, have access to adequate fitness facilities, have access to adequate medical and educational resources, have a positive life attitude, have a low adverse quotient, have few interests and hobbies, have a swallowing rage and suffering in silence and anxious and irritable personality, and often attend health training and lectures.

### Association analysis

The analysis of GLMM model revealed that ICC of the sampling area was 0.019 (95% confidence interval (CI) [−0.018–0.057], *p* = 0.228), revealing that there was no cluster effect on the sampling area. Thus, the association analysis of demographic information, lifestyle behaviors, environmental factors, and internal factors with SHS could be carried out using logistic regression.

Following the logistic regression analysis, of the 37 investigated covariates, 23 variables were found to be associated with SHS (Table 3). Regarding demographic information, the following were found to be risk factors for SHS: being older (odds ratio [OR]: 1.014, 95% CI [1.006–1.022]), a high school/junior college educational level (OR: 1.443, 95% CI [1.148–1.812]) compared with that of junior high school and below, and being divorced or widowed (OR: 1.899, 95% CI [1.313–2.747]) compared with being married; meanwhile, the following were found to be protective factors for SHS: a rural registered permanent residence (OR: 0.767, 95% CI [0.632–0.930]), a monthly household income per capita of ¥2,500 to ¥5,000 (OR: 0.767, 95% CI [0.608–0.968]) and ¥5,000 to ¥7,500 (OR: 0.731, 95% CI [0.573–0.932]), compared with an income lower than ¥2,500. Regarding lifestyle behaviors, the following were identified as risk factors for SHS: exposure to secondhand smoke (mild, OR: 1.309, 95% CI [1.052–1630]; moderate, OR: 1.465, 95% CI [1.162–1.848]; severe, OR: 1.621, 95% CI [1.262–2.083]), alcohol drinking (OR: 1.284, 95% CI [1.084–1.520]), bad eating habits (OR: 1.717, 95% CI [1.421–2.075]), not falling asleep before 11 p.m. every day (often, OR: 1.358, 95% CI [1.059–1.741]; rarely, OR:1.455, 95% CI [1.175–1.803]), and spending time online more than five hours a day (OR: 1.526, 95% CI [1.141–2.040]), compared with less than one hour a day. As to environmental factors, the following were protective factors for SHS: a good relationship with parents during one’s growth period (OR: 0.602, 95% CI [0.476–0.761]), living with good quality air (OR: 0.817, 95% CI [0.689–0.968]), living in not crowded conditions (OR: 0.636, 95% CI [0.462–0.877]), living in a harmonious neighborhood (OR: 0.775, 95% CI [0.657–0.914]), and having access to adequate fitness facilities (OR: 0.783, 95% CI [0.646–0.949]); one’s health being affected by the two-child policy (OR: 1.468, 95% CI [1.238–1.741]), and medical policies (OR: 1.265, 95% CI [1.043–1.534]) were risk factors for SHS. As for internal factors, a high adverse quotient (OR: 0.488, 95% CI [0.410–0.582]), many (≥3 kinds) interests and hobbies (OR: 0.617, 95% CI [0.520–0.733]), mature and steady personality traits (OR: 0.469, 95% CI [0.338–0.650]), compared with anxiety and irritability, high attention to one’s health (OR: 0.833, 95% CI [0.703–0.988]), and effective health promotion induced by leading a leisurely lifestyle (OR: 0.466, 95% CI [0.390–0.556]) were protective factors for SHS.

There were 12 variables associated with SHS in males (Table 3). For demographic information: area of registered permanent residence(OR: 0.699, 95% CI [0.536–0.912]) was a protective factor for SHS; an education level of high school/ junior college (OR: 1.604, 95% CI [1.190–2.163]) and university and above (OR: 1.471, 95% CI [1.058–2.046]) were risk factors for SHS. Regarding lifestyle behaviors: exposure to secondhand smoke (mild, OR: 1.383, 95% CI [1.009–1.894]; moderate, OR: 1.450, 95% CI [1.047–2.007]; severe, OR: 1.819, 95% CI [1.264–2.619]), bad eating habits (OR: 1.513, 95% CI [1.167–1.962]), not falling asleep before 11 p.m every day. (often, OR: 1.470, 95% CI [1.033–2.092]; rarely, OR: 1.564, 95% CI [1.179–2.075]) were risk factors for SHS. As to environmental factors: good relationship with parents during one’s growth period (OR: 0.600, 95% CI [0.439–0.820]) and living in crowded conditions (OR: 0.504, 95% CI [0.323–0.788]) were protective factors for SHS; having one’s health being affected by the two-child policy (OR: 1.516, 95% CI [1.203–1.910]) was a risk factors for SHS. As for internal factors: high adverse quotient (OR: 0.524, 95% CI [0.409–0.672]), many (≥3 kinds) interests and hobbies (OR: 0.592, 95% CI [0.468–0.750]), mature and steady personality traits (OR: 0.469, 95% CI [0.338–0.650]) compared with anxiety and irritability, high attention to one’s health (OR: 0.380, 95% CI [0.227–0.636]), and effective health promotion induced by leading leisurely lifestyle (OR: 0.451, 95% CI [0.353–0.578]) were protective factors for SHS.

**Table 2:** Basic descriptions of demographic information, lifestyle behaviors, environmental factors, and internal factors.

Covariates		N	%	Grouping by sex			
				male n(%)	female n(%)	*x*^2^	*p*
Age^*^		5460	41.56(16.14)	41.84(16.16)	41.29(16.11)	1.271	0.204
Body Mass Index^*^		5443	22.53(3.28)	23.21(3.18)	21.9(3.26)	15.060	<0.001
Registered Permanent Residence						0.588	0.443
	Rural area	1636	29.96	804(30.45)	832(29.5)		
	Urban area	3824	70.04	1836(69.55)	1988(70.5)		
Degree of Education						6.988	0.072
	Junior high school and below	1339	24.52	632(23.96)	707(25.07)		
	High school/ junior college	2465	45.15	1229(46.59)	1236(43.83)		
	University and above	1654	30.29	777(29.45)	877(31.1)		
Marital Status						15.182	0.001
	Married	3621	66.32	1741(66.35)	1880(66.98)		
	Unmarried	1432	26.23	732(27.9)	700(24.94)		
	Divorced or Widowed	378	6.92	151(5.75)	227(8.09)		
Monthly Household Income per Capita( ¥)						45.184	<0.001
	<2500	1282	23.48	539(20.67)	743(26.59)		
	2500-	1755	32.14	814(31.21)	941(33.68)		
	5000-	1482	27.14	782(29.98)	700(25.05)		
	7500-	467	8.55	257(9.85)	210(7.52)		
	10000-	416	7.62	216(8.28)	200(7.16)		
Smoking Status						1475.255	<0.001
	Nonsmoker	3845	70.42	1217(46.19)	2628(93.36)		
	Past smoker	535	9.80	433(16.43)	102(3.62)		
	Smoker	1070	19.60	985(37.38)	85(3.02)		
	System	10	0.18				
Exposure to Secondhand Smoke						17.618	0.001
	Never	1110	20.33	478(18.12)	632(22.41)		
	Occasionally	1655	30.31	816(30.93)	839(29.75)		
	Sometimes	1573	28.81	803(30.44)	770(27.3)		
	Often	1120	20.51	541(20.51)	579(20.53)		
	System	2	0.04				
Alcohol Consumption						771.883	<0.001
	No	2104	38.53	564(21.36)	1540(54.61)		
	Yes	3356	61.47	2076(78.64)	1280(45.39)		
Breakfast Habits						68.398	<0.001
	Everyday	2815	51.56	1232(46.7)	1583(56.15)		
	Often	1451	26.58	717(27.18)	734(26.04)		
	Sometimes	642	11.76	363(13.76)	279(9.9)		
	Occasionally	448	8.21	267(10.12)	181(6.42)		
	Never	101	3.27	59(2.24)	42(1.49)		
	System	3	0.05				
Bad Eating Habits						.776	0.378
	No	3480	63.74	1667(63.14)	1813(64.29)		
	Yes	1980	36.26	973(36.86)	1007(35.71)		
Bask in the Sun (hour/week)						52.267	<0.001
	≥7	1300	23.81	721(27.4)	579(20.62)		
	3-	1557	28.52	775(29.46)	782(27.85)		
	1-	1439	26.36	660(25.09)	779(27.74)		
	<1	1143	20.93	475(18.05)	668(23.79)		
	System	21	0.38				
Physical Exercise						40.769	<0.001
	Everyday	663	12.14	350(13.27)	313(11.1)		
	Often	600	10.99	330(12.51)	270(9.58)		
	Sometimes	1273	23.32	661(25.07)	612(21.71)		
	Occasionally	2920	53.48	1296(49.15)	1624(57.61)		
	System	4	0.07				
Falling Asleep Before 11 p.m. (day/week)						18.828	<0.001
	Every Day	1194	21.87	542(20.55)	652(23.12)		
	Often	969	17.75	426(16.15)	543(19.26)		
	Rarely	3295	60.35	1670(63.31)	1625(57.62)		
	System	2	0.04				
Spending Time Online (hour/day)						1.943	0.584
	<1	1501	27.49	705(26.7)	796(28.23)		
	1-	1374	25.16	665(25.19)	709(25.14)		
	3-	1610	29.49	796(30.15)	814(28.87)		
	≥5	975	17.86	474(17.95)	501(17.77)		
Family Structure						3.299	0.192
	Incomplete	337	6.17	162(6.19)	175(6.25)		
	Nuclear	3122	57.18	1477(56.46)	1645(58.75)		
	Extended	1957	35.84	977(37.35)	980(35)		
	System	44	0.81				
Living Together with Family						16.553	<0.001
	No	1066	19.52	575(21.85)	491(17.47)		
	Yes	4377	80.16	2057(78.15)	2320(82.53)		
	System	17	0.31				
Relationship with Parents During one’s Growth Period						3.110	0.078
	Bad	1276	23.37	644(24.47)	632(22.44)		
	Good	4172	76.41	1988(75.53)	2184(77.56)		
	System	12	0.22				
Discipline of Parents						15.726	<0.001
	Spoiled	323	5.92	156(5.94)	167(5.96)		
	Stern	1826	33.44	951(36.23)	875(31.22)		
	Positive	3279	60.05	1518(57.83)	1761(62.83)		
	System	32	0.59				
Living in Plentiful Greenery						3.871	0.049
	No	2783	50.97	1381(52.49)	1402(49.82)		
	Yes	2662	48.75	1250(47.51)	1412(50.18)		
	System	15	0.27				
Living in Good Quality Air						2.522	0.112
	No	2754	50.44	1360(51.69)	1394(49.54)		
	Yes	2691	49.29	1271(48.31)	1420(50.46)		
	System	15	0.27				
Living in Simple and Crude Houses						1.290	0.256
	Yes	730	13.37	367(13.95)	363(12.9)		
	No	4715	86.36	2264(86.05)	2451(87.1)		
	System	15	0.27				
Living in Crowded Conditions						.907	0.341
	Yes	590	10.81	296(11.25)	294(10.45)		
	No	4855	88.92	2335(88.75)	2520(89.55)		
	System	15	0.27				
Living in Harmonious Neighborhood						5.661	0.017
	No	2985	54.67	1486(56.48)	1499(53.27)		
	Yes	2460	45.05	1145(43.52)	1315(46.73)		
	System	15	0.27				
Access to Adequate Fitness Facilities						8.358	0.004
	No	4370	80.04	2154(81.87)	2216(78.75)		
	Yes	1075	19.69	477(18.13)	598(21.25)		
	System	15	0.27				
Access to Adequate Medical and Educational Resources						14.399	<0.001
	No	3384	61.98	1703(64.73)	1681(59.74)		
	Yes	2061	37.75	928(35.27)	1133(40.26)		
	System	15	0.27				
Life Attitude						4.099	0.043
	Negative	407	7.45	216(8.25)	191(6.8)		
	Positive	5017	91.89	2401(91.75)	2616(93.2)		
	System	36	0.66				
Aware of the Importance of Interpersonal Communication				.235	0.889
	Unimportance	212	3.88	103(3.91)	109(3.88)		
	General	877	16.06	418(15.86)	459(16.34)		
	Importance	4356	79.78	2115(80.24)	2241(79.78)		
	System	15	0.27				
Adverse Quotient						30.638	<0.001
	Low	2847	52.14	1274(48.57)	1573(56.08)		
	High	2581	47.27	1349(51.43)	1232(43.92)		
	System	32	0.59				
Interests and Hobbies (kinds)						51.711	<0.001
	Few (<3)	3608	66.08	1619(61.51)	1989(70.73)		
	Much (≥3)	1836	33.63	1013(38.49)	823(29.27)		
	System	16	0.29				
Personality Traits						46.087	<0.001
	Anxiety and Irritability	648	11.87	263(10.01)	385(13.77)		
	Swallowing Rage and Suffering in Silent	754	13.81	313(11.91)	441(15.77)		
	Inpatient and Aggressive	1045	19.14	502(19.11)	543(19.42)		
	Mature and Steady	2976	54.51	1549(58.96)	1427(51.04)		
	System	37	0.68				
Attending Health Training and Lectures						10.280	0.006
	Occasionally	2875	52.66	1445(54.98)	1430(50.8)		
	Sometimes	1767	32.36	824(31.35)	943(33.5)		
	Often	801	14.67	359(13.66)	442(15.7)		
	System	17	0.31				
Attention to One’s Health						3.458	0.063
	Low	3083	56.47	1524(57.88)	1559(55.38)		
	High	2365	43.32	1109(42.12)	1256(44.62)		
	System	12	0.22				
Health Promotion Induced by leading a Leisurely Lifestyle						2.485	0.115
	Moderate or Mild	4211	77.12	2054(78.55)	2157(76.76)		
	Good	1214	22.23	561(21.45)	653(23.24)		
	System	35	0.64				
Health being Affected by Two-child Policy						0.001	0.974
	No	2859	52.36	1385(54.19)	1474(54.23)		
	Yes	2415	44.23	1171(45.81)	1244(45.77)		
	System	186	3.41				
Health being Affected by medical policy						0.191	0.662
	No	3670	67.22	1786(69.79)	1884(69.24)		
	Yes	1610	29.49	773(30.21)	837(30.76)		
	System	180	3.30				

**Notes.**

* Described with mean (standard deviation),analyzed using *t* test.

**Table 3:** Multi-factor analysis of sub-health among Chinese urban residents.

Covariates		Reference group	All			Male			Female		
			*p* value	OR	95% CI	*p* value	OR	95% CI	*p* value	OR	95% CI
**Demographic Information**											
	Age		0.001	1.014	1.006–1.022						
	Registered Permanent Residence	Rural area	0.007	0.767	0.632–0.930	0.008	0.699	0.536–0.912			
	Education level	Junior school and below	0.006			0.007					
	High school/ junior college		0.002	1.443	1.148–1.812	0.002	1.604	1.190–2.163			
	University and above		0.086	1.258	0.968–1.634	0.022	1.471	1.058–2.046			
	Marital Status	Married	<0.001						0.036		
	Unmarried		0.046	1.294	1.004–1.668				0.776	1.044	0.777–1.402
	Divorced or Widowed		0.001	1.899	1.313–2.747				0.010	1.825	1.156–2.883
	Monthly household income per capita (¥)	<2500	<0.001						0.001		
	2500-		0.026	0.767	0.608–0.968				0.178	0.806	0.589–1.103
	5000-		0.012	0.731	0.573–0.932				0.002	0.599	0.433–0.827
	7500-		0.094	1.351	0.950–1.920				0.198	1.395	0.84–2.317
	10000-		0.865	1.030	0.729–1.455				0.690	0.907	0.563–1.463
**Lifestyle behaviors**											
	Exposure to Secondhand Smoke	None	0.001			0.012					
	Mild		0.016	1.309	1.052–1.630	0.044	1.383	1.009–1.894			
	Moderate		0.001	1.465	1.162–1.848	0.025	1.450	1.047–2.007			
	Severe		<0.001	1.621	1.262–2.083	0.001	1.819	1.264–2.619			
	Alcohol Consumption	No	0.004	1.284	1.084–1.520				0.008	1.374	1.087–1.739
	Bad Eating Habits	No	<0.001	1.717	1.421–2.075	0.002	1.513	1.167–1.962	<0.001	2.068	1.580–2.707
	Falling Asleep Before 11 p.m. (day/week)	Every Day	0.002			0.007					
	Often		0.016	1.358	1.059–1.741	0.032	1.470	1.033–2.092			
	Rarely		0.001	1.455	1.175–1.803	0.002	1.564	1.179–2.075			
	Spending Time Online (Hour/Day)	<1	0.030						0.017		
	1-		0.547	1.073	0.853–1.35				0.714	0.944	0.694–1.284
	3-		0.267	1.144	0.902–1.451				0.706	0.942	0.691–1.285
	≥5		0.004	1.526	1.141–2.040				0.013	1.668	1.114–2.498
**Environmental factors**											
	Relationship with Parents During Growth Period	Bad	<0.001	0.602	0.476–0.761	0.001	0.600	0.439–0.820	<0.001	0.512	0.362–0.724
	Living in Good Quality Air	No	0.019	0.817	0.689–0.968				0.008	0.723	0.569–0.918
	Living in Crowded Conditions	Yes	0.006	0.636	0.462–0.877	0.003	0.504	0.323–0.788			
	Harmonious Neighborhood	No	0.002	0.775	0.657–0.914				0.001	0.668	0.530–0.842
	Adequate Fitness Facilities	No	0.012	0.783	0.646–0.949				0.028	0.749	0.579–0.970
	Health Being Affected by Two-child Policy	No	<0.001	1.468	1.238–1.741	<0.001	1.516	1.203–1.910	<0.001	1.629	1.294—2.050
	Health Being Affected by medical policy	No	0.017	1.265	1.043–1.534						
**Internal factors**											
	Adverse Quotient	Low	<0.001	0.488	0.410–0.582	<0.001	0.524	0.409–0.672	<0.001	0.436	0.342–0.554
	Interests and Hobbies (kinds)	Few (<3)	<0.001	0.617	0.520–0.733	<0.001	0.592	0.468–0.750	<0.001	0.621	0.486–0.792
	Personality Traits	Anxiety and Irritability	<0.001			<0.001			<0.001		
	Swallowing Rage and Suffering in Silent		0.918	0.979	0.648–1.479	0.732	0.892	0.464–1.715	0.853	0.951	0.559–1.619
	Inpatient and Aggressive		0.057	0.700	0.484–1.011	0.072	0.592	0.335–1.048	0.226	0.743	0.459–1.202
	Mature and Steady		<0.001	0.469	0.338–0.650	<0.001	0.380	0.227–0.636	0.002	0.519	0.340–0.791
	Attention to one’s health	Low	0.036	0.833	0.703–0.988						
	Health Promotion Induced by Leading a Leisurely Lifestyle	Moderate or Mild	<0.001	0.466	0.390–0.556	<0.001	0.451	0.353–0.578	<0.001	0.424	0.333–0.540

There were 14 variables associated with SHS in females (Table 3). For demographic information: being divorced or widowed (OR: 1.825, 95% CI [1.156–2.883]) was a risk factor for SHS, compared with being married; meanwhile, a ¥5000 to ¥7500 monthly household income per capita (OR: 0.599, 95% CI [0.433–0.827]) was a protective factor for SH, compared with an income lower than ¥2500. Regarding lifestyle behaviors: alcohol drinking (OR: 1.374, 95% CI [1.087–1.739]), bad eating habits (OR: 2.068, 95% CI [1.580–2.707]), and spending time online more than five hours a day (OR: 1.668, 95% CI [1.114–2.498]) compared with less than one hour a day were risk factors for SHS. As to environmental factors: good relationship with parents during one’s growth period (OR: 0.512, 95% CI [0.362–0.724]), living with good quality air (OR: 0.723, 95% CI [0.569–0.918]), living in a harmonious neighborhood (OR: 0.668, 95% CI [0.530–0.842]), and having accesses to adequate fitness facilities (OR: 0.749, 95% CI [0.579–0.970]) were protective factors for SHS; one’s health being affected by the two-child policy (OR: 1.629, 95% CI [1.294–2.050]) were risk factors to SHS. As for internal factors: high adverse quotient (OR: 0.436, 95% CI [0.342–0.554]), many (≥3 kinds) interests and hobbies (OR: 0.621, 95% CI [0.486–0.792]), mature and steady personality traits (OR: 0.519, 95% CI [0.340–0.791]) compared with anxiety and irritability, and effective health promotion induced by leading leisurely lifestyle (OR: 0.424, 95% CI [0.333–0.540]) were protective factors for SHS.

## Discussion

This study was the first to systematically investigate the influencing factors of SHS from the aspects of demographic information, lifestyle behaviors, environmental factors and internal factors with a sample of Chinese urban residents at the national level. A total of 23 variables were significantly associated with SHS, of which 12 were significantly associated with males and 14 with females. All these variables that were significantly associated with SHS were from demographic information, lifestyle behaviors, environmental factors and internal factors, which thereby indicates that the association factors were widespread, not only demographic information and external factors, such as lifestyle behaviors and environmental factors, but also internal factors.

Regarding demographic information, age was significantly associated with SHS in all participants. However, association with SHS was not found between men or women. For all urban participants, especially males, whose registered permanent residence was in an urban area, it was found that there people were not easily prone to suboptimal health. This finding was in line with the actual real-life situation in society that rural–urban migrant workers have greater life stress and work stress than do urban-native residents (Cui et al., 2012). Education level was found to be significantly associated with SHS in all participants, especially in males, with having a higher education level leading to an easier chance of developing SHS. This might be due to high-pressure jobs and working longer hours than people with lower educational levels (Schieman & Glavin, 2011). Women who ate divorced and widowed have been reported to have a 82.5% higher risk of developing SHS compared with married women. Compared with men, divorced women have an increased risk of living in poverty and being a single parent (Leopold, 2018); being widowed may severely restrict women’s ability to access financial, affective, informational, or physical resources, which in turn may affect health outcomes (Perkins et al., 2016). Previous studies have reported a significant association between income and health. Studies in the United States have pointed out that the effect of income on health tapers off at around the median level of income (Knaul et al., 2006; Arsenijevic, Pavlova & Groot, 2013). Our findings are in line with these studies in the United States. We also found that women whose monthly household income per capita was between ¥5,000 and ¥7,500 were not prone to developing SHS compared with those whose income was less than ¥2,500.

Concerning lifestyle behaviors: The impacts on health exerted by secondhand smoke are self-evident (Iloh, 2017). In the present study we investigated the same association of exposure to secondhand smoke with suboptimal health in males. The risk of detection rate of suboptimal health was increased when exposure to secondhand smoke was more severe. Alcohol drinking also plays a significant role in the etiology of many acute (Rehm et al., 2010) and chronic diseases, and can cause a major burden to the population’s health ,accounting for 4% of disability adjusted life years worldwide (Whiteford et al., 2013). Alcohol consumption can increase the risk of SHS by 37.4% in females. However, no significant association was found between alcohol consumption and SHS in men. This difference between males and females may be explained by the cause for drinking. Drinking in Chinese culture can be classified mainly as social drinking, which is an actively usually widely accepted in men than women (Cochrane, 2003). In our study, we found that bad eating habits were associated with SHS in both males and in females. These bad eating habits that were investigated included irregular eating, poor dieting, overeating, partial or picky eating, high intake of salt, eating spicy food, and usually eating snacks instead of regular meals and etc. Eating salty food is not good for the health and may increase the risk of incidence of stomach cancer (Park et al., 2016). While having an unhealthy diet or overeating can induce heavy gastrointestinal burden and go against one’s overall health (Janssen, 2010). Meanwhile, spending a prolonged amount of time online may cause backache, neck, finger, wrist, and arm pain, as well as emotional fluctuation, tension and anxiety, fatigue and other SHS symptoms (Karacic & Oreskovic, 2017). In traditional medicine, the time past 11 p.m. is considered to be the time for organ detoxification, and a need for deep sleeping (Li & Li, 2017). Going to bed before 11 p.m. and having enough hours of sleep are important for the strengthening of the body’s resistance and immunity (Chen, 2012). We investigated whether there is a significant association with falling asleep before 11 p.m. and SHS, and for those who can’t could not fall asleep before 11 p.m. whether these people had a higher risk of developing SHS. We found that spending time more than five hours a day online increased the risk of SHS by 66.8%.

As to environmental factors, males and females with good relationships with their parents during their growth period were less susceptible to SHS, which is consistent with the findings of several previous studies (Xue et al., 2019a; Xue et al., 2019b; Xue et al., 2019c). More attention should be paid to the relationship between parents and children when proceeding with family education to allow for the protections of children’s health and growth. Good air quality is closely associated with optimal health, and the present study showed that women who live with good air have a lower risk of attaining suboptimal healthy. However, this association was not found in males. Living in crowded conditions showed negative association with SHS in males, but not in females. This might be because living in crowded conditions comes with foul air, disturbing noise, and tension, which impose strains and harm to people’s physical and mental health (Gibson et al., 2011). We found that living in a harmonious neighborhood and having access to adequate fitness facilities were all associated with SHS in females, not in males. Harmonious neighborhoods brings with them a safe and mutually supportive living environment, which is good for both physical and mental health. A study has shown that higher perceived neighborhood disorder was significantly associated with a higher level of total health service usage among those with lifestyle illnesses (Martin-Storey et al., 2012). The popularization of community fitness facilities has provided residents with convenience in accessing and undertaking fitness programs and helps to ease residents to participate in fitness programs, especially for those with weak fitness awareness; the popularization of such facilities is an important component of the *Healthy China 2030* plan (Li, 2018). Both male and female patients whose health was affected by the two-child policy had higher risk of developing SHS. A major concern after the implementation of two-child policy has been the acute shortage of pediatricians and paediatric nurses in China, which has worsened over the past decade (Han, Hu & Wang, 2014). Therefore, for parents with lower incomes and who work in high-pressure working conditions, the demand for a newborn child will inevitably bring more anxiety which is not good for the health (Ren et al., 2015).

Lastly, on internal factors, adverse quotient,( also known as setback business or counter business), refers to people’s ability to resolve and withstand setbacks, usually the more one is able to bear such setbacks, the higher the adverse quotient is (Zhao, 2016). In our research team’s previous study, it was found that the higher the adverse quotient, the lower the risk of suboptimal health (Xue et al., 2019a; Xue et al., 2019b; Xue et al., 2019c). In the present study, we found that a high adverse quotient was a protective factor against SHS in both males and females. Participants (both males and females) with more than three kinds of interests and hobbies had a lower risk of developing SHS compared with those with less than three interests and hobbies. People with a wide range of hobbies have been reported to be less likely to have mental health problems, and their physical to be better than those with less interests and hobbies (Hirosaki et al., 2009). Furthermore, the findings of our group’s previous research indicated that the personality and SHS were related, and that those urban residents with a personality pertaining to swallowing rage and suffer in silence, inpatience and aggressiveness have significantly lower scores than those of urban residents with a mature and steady character (Xue et al., 2019a; Xue et al., 2019b; Xue et al., 2019c). Hence, the development of a mature and steady personality may have a positive effect for improving health. This study also exhibited the same findings in both males and females.

The protective effect of high attention to one’s health with SHS was found in all participants, not between females nor in males. The protective effect of effective health promotion induced by leading a leisurely lifestyle with SHS was found in both males and females. The forms of leisure and its corresponding effects are of great significance for fatigue reduction and health recovery (Kubo et al., 2013). Proper recreational activities were contributed to health promotion, and enhance the quality of life and health outcomes.

### Limitations

This study has several limitations. First, although we used face-to-face interviews, all data were collected from a respondent-completed questionnaire; thus, responses may have a level of inherent inaccuracy or bias. Second, although we used a four-stage stratified sampling method, sampling errors were still inevitable. Lastly, this study can only find correlation, not causal relationship, due to its cross-sectional study rather than longitudinal approach.

## Conclusions

Through this large cross-sectional study of Chinese urban residents, we found that SHS was significantly associated with demographic information, lifestyle behaviors, environmental factors and internal factors, both in males and females. Our study supports propositions regarding the benefits of controlling both internal and external factors in preventing suboptimal health.

##  Supplemental Information

10.7717/peerj.10508/supp-1 Supplemental Information 1 Descriptive data for five provinces and cities

## Additional Information and Declarations

### Data Availability

The following information was supplied regarding data availability: Raw data is available as a Supplemental File.
